# The REGIA Database (RegiaDB): Status, Limitations and Future Developments

**DOI:** 10.1002/cfg.144

**Published:** 2002-04

**Authors:** Ramon Alonso-Allende, José M. Fernández-González, Osvaldo Graña, Alfonso Valencia

**Affiliations:** Protein Design Group National Center for Biotechnology CNB-CSIC Cantoblanco, Madrid E-28049 Spain


**Comparative and Functional Genomics**
*Comp Funct Genom* 2002; **3**: 109–114.
Published online 1 March 2002 in Wiley InterScience (www.interscience.wiley.com). **DOI:** 10.1002/cfg.144

**Conference Review**

### The REGIA database (RegiaDB): status, limitations and future developments

Ramon Alonso-Allende, José M. Fernández-González, Osvaldo Graña and Alfonso Valencia*
*Protein Design Group, National Center for Biotechnology, CNB-CSIC,Cantoblanco, Madrid E-28049, Spain*

**Correspondence to:*
*Protein Design Group, National Center for Biotechnology, CNB-CSIC,Cantoblanco, Madrid 28049, Spain.*
*E-mail: valencia@cnb.uam.es*

**Keywords: database schema; transcription factors; *Arabidopsis*; relational database; query systems**

Received: 2 January 2002
Accepted: 29 January 2002

### The REGIA project

REGIA (the REgulatory Gene Initiative in *Arabidopsis*) is financed by the European Union (EU) (QLG2-1999-00876) and carried out by a consortium of 29 laboratories in 9 EU countries (**http://www.epsoweb.org/catalog/EU/fp5/REGIA.htm**).

The objective of this project is the functional characterization of transcription factors (TFs) from *Arabidopsis*, to obtain insight into their evolution, and the regulatory hierarchies, gene redundancies, and functional interdependencies that exist among TFs. Such interdependencies are of pivotal importance, as TFs rarely act alone in controlling expression of their target genes. Most traits involve a large number of genes, which are controlled by a few TFs. An understanding of the role of TFs should therefore facilitate the manipulation of agronomically important traits in crop plants such as tomato or maize. This may lead to plants that are more resistant to a variety of stresses, such as, drought, disease or ultra-violet light.

Approaches being used to achieve this objective are:

1. Analysis of expression patterns of known TFs (DNA array experiments)
2. Identification of mutations in a large number of TF loci
3. Ectopic expression of selected TFs genes in *Arabidopsis* and crop plants
4. Phenotypic and metabolic analysis of plants harbouring mutations at TF loci or overexpressing TFs
5. Analysis of interactions between TFs using the two-hybrid screen (2HS).
6. Implementation of a computational infrastructure.

These actions would be impossible to complete without this international collaboration and its scientific co-ordination by Dr. Javier Paz-Ares.

The work is distributed among the members of the consortium based upon their expertise and particular interest in certain TFs. Each of the 28 experimental groups is responsible for cloning, generating mutants, creating transgenic plants and analysing the phenotype of a set of 49 TFs. Five groups are also in charge of the co-ordination of the experimental actions (Figure 1). In the case of the 2HS and metabolic analysis, the co-ordinating groups will carry out the experiments, while in the case of the DNA array the co-ordinating group will mainly prepare the arrays and each group will develop the experiments on their own.

The information about the preparation conditions of the TFs, preparation of the experiments and the final results will be stored and managed by our group (Protein Design Group, CNB-CSIC), which acts as the bioinformatics co-ordinator of the project. In order to cope with the vast quantity and diversity of information we expect to be generated

Copyright © 2002 John Wiley & Sons, Ltd.

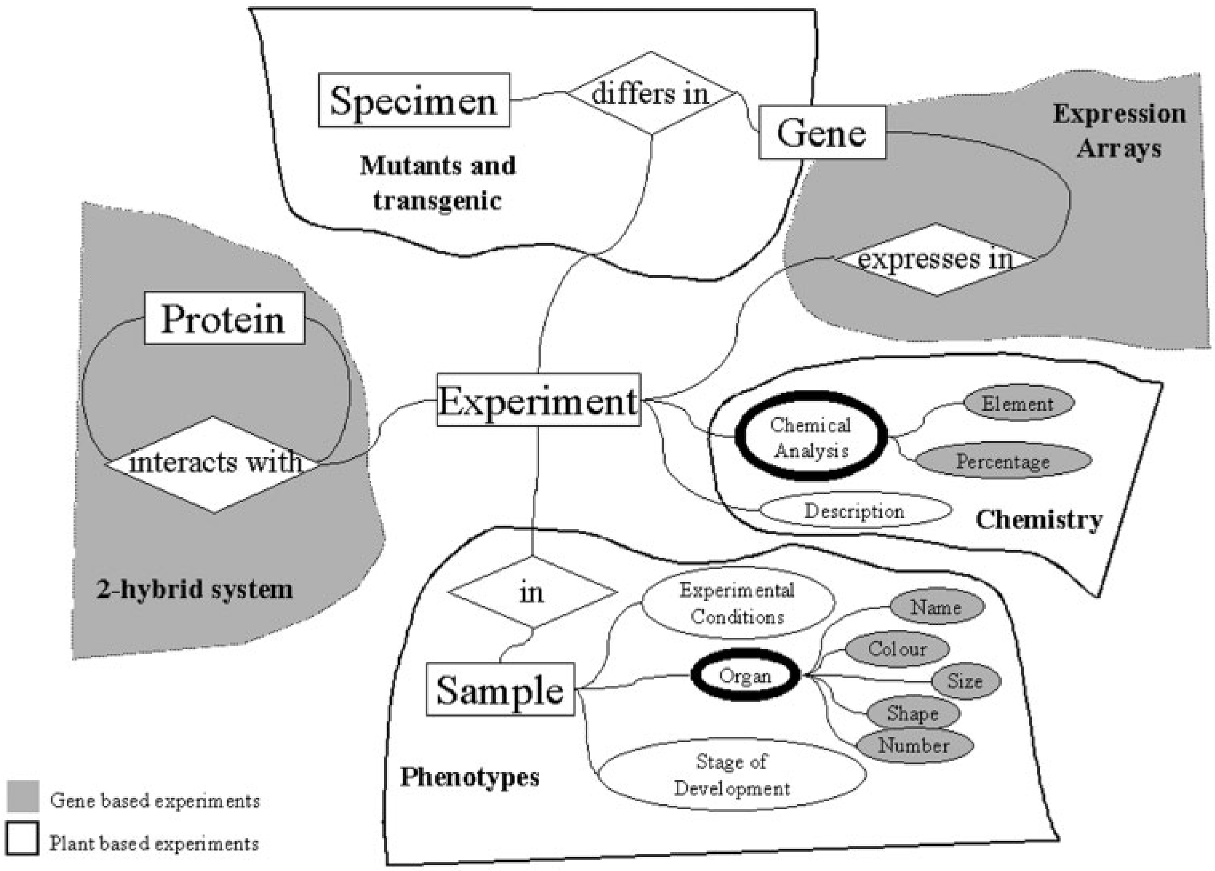

**Figure 1.** Relational schema of the 5 experimental areas. Each area has a co-ordinator in charge of the follow up of the area in the project. The RegiaDB is based on this schema to store the experimental data in a relational manner by the project, we have designed and implemented a robust relational database schema and a comprehensive analysis system, which will allow not only the ordered storage of data, but also the formulation of complex queries of the results by a group of experimental biologists.

### The REGIA relational database schema

The basic warehouse system has been designed as a classical relational database with its corresponding SQL querying interface. The database schema is complex enough to store not only the different experimental results but also the output of different bioinformatic operations, e.g. BLAST searches (**http://www.ncbi.nlm.nih.gov/BLAST/**) for similar sequences or similarity of expression profiles. The current implementation runs under a PostgreSQL managing system. Even if the relational schema is the most obvious technical solution that can be implemented in the time frame of the project, we are well aware of its limitations for dealing with the type of data discussed here.

### Preparation of the design of RegiaDB

Before designing the database, we interviewed a number of experimental biologists to identify their requirements. It became clear that the design of RegiaDB would be a major advance for many of them and it can be considered as a first prototype for entering in the new possibilities offered by the post-genomic technologies.

The database schema was designed during the first six months of the project, and revised as we learned the details of various experimental approaches (Figure 1). For example, more than nine months after initiating the design we discovered

Copyright © 2002 John Wiley & Sons, Ltd. 

that transgenic plants could include a number of mutated genes, and there was no one-to-one relation between mutant genes and phenotypes and metabolic profiles. This fact could have been detected earlier only if the experimental biologists could have been more active during the design of the database. The incorporation of this apparently trivial change required a re-designed database schema moving from a gene-centric approach to one based on plants (and genome composition). Obviously it would have been better to design a complete schema before starting the experimental part of the project, the real constraints have made us to adapt to the changing requirements of the project, a situation that is probably not unique to this project.

### Interrogation levels in RegiaDB

The current implementation of RegiaDB will enable the formulation of questions at different levels of complexity and with different implications in terms of database requirements.

The first set of questions corresponds to simple relations that can be directly derived from the database and do not require the application of bioinformatic tools (Box 1).

### Box 1. Basic questions, directly addressing information contained in RegiaDB

- Retrieve information (names, intron/exon structure with a reliability score, sequence, protein, Accession numbers in other databases) on all the Arabidopsis genes analysed in REGIA.
- Select information on proteins (names, gene, sequence, Accession numbers in other databases) belonging to a certain group (REGIA partner).
- List mutated genes belonging to a certain family of TF.
- How many transgenic crop plants have been constructed?
- List those genes expressed in a certain experimental condition.
- Retrieve the metabolic profiles of the mutants of two known interacting TFs.
- List those genes whose mutation affects flowering.
- Find the gene locations of interacting proteins.

Answering the second type of questions requires the combination of the basic information stored in RegiaDB with the results of external methods (Box 2).

### Box 2. Questions that depend on the results of external methods

- Which is the most abundant sequence domain present in a TF combined with a certain DNA binding domain and conserved in other species? To answer this requires the incorporation of an external method for searching TF sequences for PFAM profiles (**http://www.sanger.ac.uk/Software/Pfam/index.shtml**).
- Which of the proteins in a certain TF family have a known three-dimensional structure homologue? This requires a BLAST search against the protein structure database with all protein sequences in RegiaDB assigned to that TF family.
- Retrieve all genes with an expression pattern similar to the one of gene X. The answer requires the application of a clustering technique on the DNA array profiles stored in RegiaDB.
- Which TFs interact with a given TF, and have a similar expression pattern but produce different metabolites in transgenic plants? This requires the external comparison of expression and metabolic profiles.

Technically the analysis tools have to be integrated into the database so that they are able to extract data from it and insert their results into it. RegiaDB has been designed to allow the integration of results from different sequence searching techniques, DNA array clustering results and comparisons of metabolic profiles.

Finally, there is a type of complex question that would require access to RegiaDB, followed by access to external analysis systems that are not directly integrated into the database (Box 3). These questions cannot be solved in the frame of RegiaDB, which can only provide input data for a more complex, expert based study.

Copyright © 2002 John Wiley & Sons, Ltd. 

### Box 3. Complex queries that require access to external methods

- Build a phylogenetic tree of all the TFs that belong to a certain family and have been identified in complete eukaryotic genomes. This can be solved, in a first approach, by collecting all the similar sequences in other organisms with BLAST, automatically aligning them and building the corresponding phylogenetic tree. It would require a detailed human-expert analysis of the results.
- How can a mutation of a TF affect the structure of the protein? There is no simple solution to this question. What RegiaDB could provide is the three dimensional representation of the protein (or a model by homology), automatically highlighting the position of the mutation.
- Retrieve enzymes whose activity in metabolism can be affected by the mutation of a TF. In principle, it is possible to deduce the enzymes related to the alteration of metabolites produced in a given transgenic plant by interrogating a database such as KEGG (**http://www.genome.ad.jp/kegg/kegg.html**).

In this case the query, that has to be carried out externally to RegiaDB, would provide the list of metabolites altered in the transgenic plant produced with the specific TF.

### RegiaDB interface and access

The interaction of the members of the consortium is regulated by the structure of the REGIA project. All the groups have access to the group of genes they are directly responsible for and to the experimental results they have generated. Only the coordinators of some of the working groups, i.e. transcription factor probes, expression patterns, analysis of interactions, metabolic profiles and crop transformation, can input information regarding the experiments they are in charge of. All the groups will have access to the final integrated results.

This data property structure is more complicated than those provided by current database manager systems (e.g., PostgreSQL). In RegiaDB each entry is linked to an internal table where reading and updating data properties are stored for every user of the database, and an explicit data property schema has been established for the relations between entities with data belonging to different partners.

All partners interact with the database in four ways:

1. Input of the basic experimental data: Web forms have been developed to facilitate the distribution of data concerning the biological material in a standard format, i.e. transcription factor genes cloned in different vectors for the DNA arrays, Y2H and metabolic experiments.
2. Results of experiments: The results from the centralised experiments are sent back to those responsible for the particular genes, at the same time they are stored in RegiaDB through the net.
3. Bioinformatic analysis of REGIA data: A set of tools for bioinformatic analysis has been implemented as part of the database, which can be accessed through web specific forms. It is currently possible to carry out BLAST and hidden Markov model sequence searches, and to visualize protein interactions with viewers based on Java technology (an example can be seen at: **http://www.pdg.cnb.uam.es/suiseki/system/Start_e2f.html**). We are in process of implementing tools for the comparison of metabolic profiles and analysis and clustering of expression arrays.
4. Data browsing. The REGIA groups, and in the future any user, will be able to access the data through the web by a related system of links, pre-stored views of the data and a simplified graphical SQL system (Figure 2).

As it is always difficult to consult relational database system, we are developing a user-friendly interface. Changes in the schema involve new development environment for the Web interface and modifications in the input control data and query system.

### Problems encountered during the design and implementation of RegiaDB

### Heterogeneous data

RegiaDB stores data from different types of experiments, stored in other external databases, and produced by a set of computational methods.

Copyright © 2002 John Wiley & Sons, Ltd.

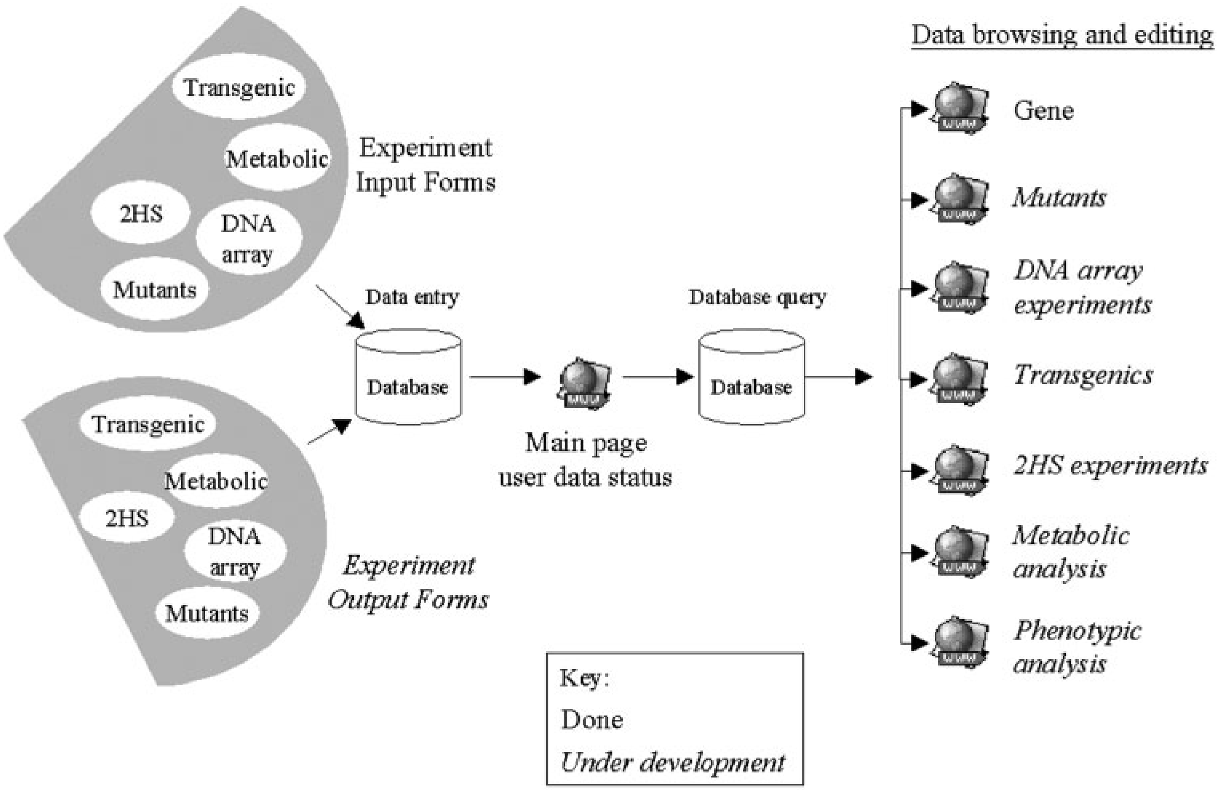

**Figure 2.** Schema of the RegiaDB Web interface

Combining them into a single schema requires a considerable level of integration and resolution of data dependencies. Consequently, changes in the structure of the data sources are difficult to incorporate and will decrease the stability of the database. To avoid some of these problems we have embedded RegiaDB in an XML structure that simplifies the interface and insertion of external data sources.

### Collaborating with experimental biologists

As mentioned above, it is difficult to build a system at the same time as the experiments are being carried out, even more so in the absence of well-established field experience in the combination of post-genomics data. During this process we have also realised how difficult it is to explain to experimental biologists the importance of the database structure for their work, the effort required in order to develop such a complex schema or how difficult it is to incorporate changes to the data structure after a certain point. Ultimately, we have realised the difficulty of encouraging their active participation in the development of the database.

### Lack of standards for different data types

For experiments such as protein interaction detection, and phenotypic and metabolic analyses there are no available data standards, and even for expression data a common format is still under discussion.

This lack of standards has had a negative influence on the design of RegiaDB, which contains a considerable set of fields with non-systematic free-text, particularly in the description of the experimental techniques used for the cloning of genes.

It is important to keep in mind that RegiaDB has incorporated decisions about data formats that will determine its future possibilities of portability and integration with other databases.

### Extraction of information

In general, relational databases are not user-friendly. Basic computational skills and knowledge

Copyright © 2002 John Wiley & Sons, Ltd. 

of the database structure are needed for extracting the stored information via SQL queries. To make RegiaDB useful to the partners we are developing direct views of the data that will correspond to the more commonly asked questions, and a graphical query system that will incorporate the main capabilities of the SQL system and its adaptation to the RegiaDB data structure.

### Acknowledgement

We acknowledge the help of JC Oliveros-Collazos for the construction of the expression array related schema and of Armando Amat for the maintenance of the system. The help and support of other members of the Protein Design Group is also deeply acknowledged. Different members of the REGIA consortium have provided us with insightful comments about the type of experimental results, and with an interesting set of questions to be addressed by the system, of which only a small part is presented here.

RAA and JMFG worked on the design of the DB schema. RAA and OG are responsible for the web infrastructure for data insertion and retrieval, and have also incorporated different variations to the database. JMFG developed the data property system and the XML structure around the database (required for future interaction with other databases). AV has coordinated the project and adopted the main decisions about the type of data that would be integrated in the database.

The work has been financed by grant QLG2CT1999-00876 of the EU, and bio2000-2057-CE from the Spanish government.

Copyright © 2002 John Wiley & Sons, Ltd.

